# The variome of pneumococcal virulence factors and regulators

**DOI:** 10.1186/s12864-017-4376-0

**Published:** 2018-01-03

**Authors:** Gustavo Gámez, Andrés Castro, Alejandro Gómez-Mejia, Mauricio Gallego, Alejandro Bedoya, Mauricio Camargo, Sven Hammerschmidt

**Affiliations:** 1 Genetics, Regeneration and Cancer Research Group (GRC), University Research Centre (SIU), Universidad de Antioquia (UdeA), Calle 70 # 52 - 21, 050010 Medellín, Colombia; 2 Basic and Applied Microbiology Research Group (MICROBA), School of Microbiology, Universidad de Antioquia (UdeA), Calle 70 # 52 - 21, 050010 Medellín, Colombia; 3 Department of Molecular Genetics and Infection Biology, Interfaculty Institute of Genetics and Functional Genomics, Center for Functional Genomics of Microbes, Ernst-Moritz-Arndt University of Greifswald, Felix-Hausdorff-Str. 8, D-17487 Greifswald, Germany; 4 School of Microbiology, University of Antioquia, Bloque 5 - Oficina 408, Calle 70 # 52 - 21, 050010 Medellín, Colombia

**Keywords:** Variome, Virulence factors, Two component systems, *Streptococcus Pneumoniae*

## Abstract

**Background:**

In recent years, the idea of a highly immunogenic protein-based vaccine to combat *Streptococcus pneumoniae* and its severe invasive infectious diseases has gained considerable interest. However, the target proteins to be included in a vaccine formulation have to accomplish several genetic and immunological characteristics, (such as conservation, distribution, immunogenicity and protective effect), in order to ensure its suitability and effectiveness. This study aimed to get comprehensive insights into the genomic organization, population distribution and genetic conservation of all pneumococcal surface-exposed proteins, genetic regulators and other virulence factors, whose important function and role in pathogenesis has been demonstrated or hypothesized.

**Results:**

After retrieving the complete set of DNA and protein sequences reported in the databases GenBank, KEGG, VFDB, P2CS and Uniprot for pneumococcal strains whose genomes have been fully sequenced and annotated, a comprehensive bioinformatic analysis and systematic comparison has been performed for each virulence factor, stand-alone regulator and two-component regulatory system (TCS) encoded in the pan-genome of *S. pneumoniae*. A total of 25 *S. pneumoniae* strains, representing different pneumococcal phylogenetic lineages and serotypes, were considered. A set of 92 different genes and proteins were identified, classified and studied to construct a pan-genomic variability map (variome) for *S. pneumoniae*. Both, pneumococcal virulence factors and regulatory genes, were well-distributed in the pneumococcal genome and exhibited a conserved feature of genome organization, where replication and transcription are co-oriented. The analysis of the population distribution for each gene and protein showed that 49 of them are part of the core genome in pneumococci, while 43 belong to the accessory-genome. Estimating the genetic variability revealed that pneumolysin, enolase and Usp45 (SP_2216 in *S. p.* TIGR4) are the pneumococcal virulence factors with the highest conservation, while TCS08, TCS05, and TCS02 represent the most conserved pneumococcal genetic regulators.

**Conclusions:**

The results identified well-distributed and highly conserved pneumococcal virulence factors as well as regulators, representing promising candidates for a new generation of serotype-independent protein-based vaccine(s) to combat pneumococcal infections.

## Background

*Streptococcus pneumoniae*, also known as the pneumococcus, is a Gram-positive, α-hemolytic and facultative aerobic bacterium. This microorganism is normally found as a harmless commensal in the upper respiratory tract of humans. Pneumococi have a great epidemiological importance due to their high impact on public health, causing more than one and a half million of deaths per year around the world [1]. *S. pneumoniae* is the main etiologic agent of community-acquired pneumonia. However, this is not its only clinical manifestation, because other kind of diseases such as otitis media, sinusitis, septicemia and meningitis are also caused by this pathogen and associated with high mortality rates [2].

Given the particular biochemical and molecular features of *Streptococcus pneumoniae* (Gram-positive, catalase-negative, optochin-sensitive and bile-soluble bacteria), its identification process in the laboratory is relatively simple. Nevertheless, the great molecular, biochemical and immunological diversity of its capsule and other antigens such as choline-binding proteins make them one of the hardest bacterial pathogens to face because of its variability [3, 4]. The “*Quellung Reaction*”, developed over 100 years ago by Neufeld, allows the specifical and reliable identification of each one of the >94 serotypes that have been discovered up to date. The capsular polysaccharide is the sine qua non virulence factor, however the pathogenic potential of serotypes may vary and similarly, the frequencies or prevalence varies from one geographic region to the other [5]. Despite this, the capsule is not the only factor required to induce disease by *S*. *pneumoniae.* In fact, the surface of the pneumococcus is decorated by various proteins, which have been already associated with its high pathogenic potential. In addition, their interaction level with the host cellular receptors has been proved, exhibiting crucial pathogenic functions such as adhesion, colonization, breaching tissue barriers and immune evasion [6].

An important group of regulatory proteins of great interest are the histidine kinases (HK), located in the bacterial surface and functioning as the sensors of two-component regulatory systems (TCS). The sensing of environmental signals via TCS, regulates the genetic expression of cellular processes that are of great importance such as natural competence, antibiotic resistance, adaptation to different environmental situations, surface proteins expression, and others [7, 8]. In general, TCS are composed of a histidine kinase, a membrane protein sensing the extracellular signals and transmitting these signals to a cytoplasmatic regulator/effector protein refered to as response regulator (RR). This happens via the HK autophosphorylation and a subsequent trans-phosphorylation process. In *Streptococcus pneumoniae,* 13 TCS and one orphan RR have been identified [7].

The relevance of the cellular, physiological and pathogenic functions that these pneumococcal proteins fulfill, have aroused a great scientific and biotechnological interest, given their potential pharmaceutical applications as vaccine candidates [9]. Nowadays, the antibiotic treatment of the infections caused by the pneumococcus is often complicated due to the increase of antibiotic resistance [10]. Furthermore, prevention by the use of the pneumococcal polysaccharide vaccines and/or pneumococcal conjugated vaccines only helps to control the disease caused by some of the serotypes and has an indirect impact on colonization [9]. Thus, there is an urge to define more global and effective strategies for the treatment and/or prevention, and to fight the pneumococcus and its local and invasive diseases. Consequently, the idea of a protein-based vaccine has taken great importance in the last years. However, in order to be considered or included in a recombinant vaccine formulation, a bacterial protein has to fulfill specific criteria such as: (1) playing an important role in the bacterial fitness and/or pathogenesis of *S. pneumoniae*, (2) possessing a wide distribution among the circulating strains and clinical isolates, (3) exhibiting a major conservation at its genetic and protein sequence, (4) being inmunogenic, (5) demonstrating protectivity in experimental assays, and (6) having favorable physico-chemical properties for expression and purification of its recombinant products.

*Streptococcus pneumoniae* is a pathogen exhibiting a fratricide behavior and an enormous capacity for natural competence, acquiring foreign genetic material and integrating it into its genome [11]. These processes, in addition to the mutation rates [12, 13], greatly stimulate the horizontal gene transfer with other microorganisms, and explains pneumococcal genetic variability and genome plasticity [14, 15]. This model of pneumococcal population evolution, where recombination highly outpasses mutation, is also caused by the relatively high numbers of repetitive sequences in the genome thereby facilitating the incorporation of foreign DNA in the chromosome [15–18]. In consequence, these events contribute to structural reorganizations, and influence the presence or absence of protein-encoding genes in differente subsets of the global pneumococcal population, making them highly heterogeneous from the core- and pan-genomic point of view [15]. Likewise, the generation and fixation of particular changes in the genome affect the mutation rates, which in turn influence the evolution and conservation of genes and contribute to adaptative changes that potentially lead to an increased virulence and a more complex interaction with the host [19].

Due to these molecular events and their importance, there is a need to fully and globally understand the genetic heterogeneity and variability among the different pneumococcal strains/serotypes (variome), and to get a deeper and detailed molecular undestanding of the different physiological and pathogenic mechanisms that this microorganim uses to cause severe and life-threatening diseases. Definitely, obtaining this knowledge will allow to identify potential pharmaceutical targets for new antimicriobial therapies. By the recognizition of their conservation and distribution degree among pneumococcal strains, this will confirm protein candidates for vaccines. However, despite the availability of a high number of completely sequenced genomes and the importance to analyse the genetic differences among pneumococci, only a few studies have focused on studying its variability from a global perspective, similarly as the Human variome databases do [20]. To date only the “Microbial Variome Database” [21], which possesses and organizes the available information of the variome of the two Gram-negative bacterial species *Escherichia coli* and *Salmonella enterica*, is providing such information for microorganisms. Remarkably, there are no open-source data of this nature for any Gram-positive bacterial genome. Hence, this study focused on the construction of the first *S. pneumoniae* Variome model, starting with the identification of all allellic and protein variants, a mutation and distribution analysis (presence and absence) of the virulence factors and regulators, among a set of pneumococcal strains that possess a fully sequenced and annotated genome.

## Methods

### Definition of the study population set and determination of the optimal representation of the entire population of pneumococci

**Fig. 1:**
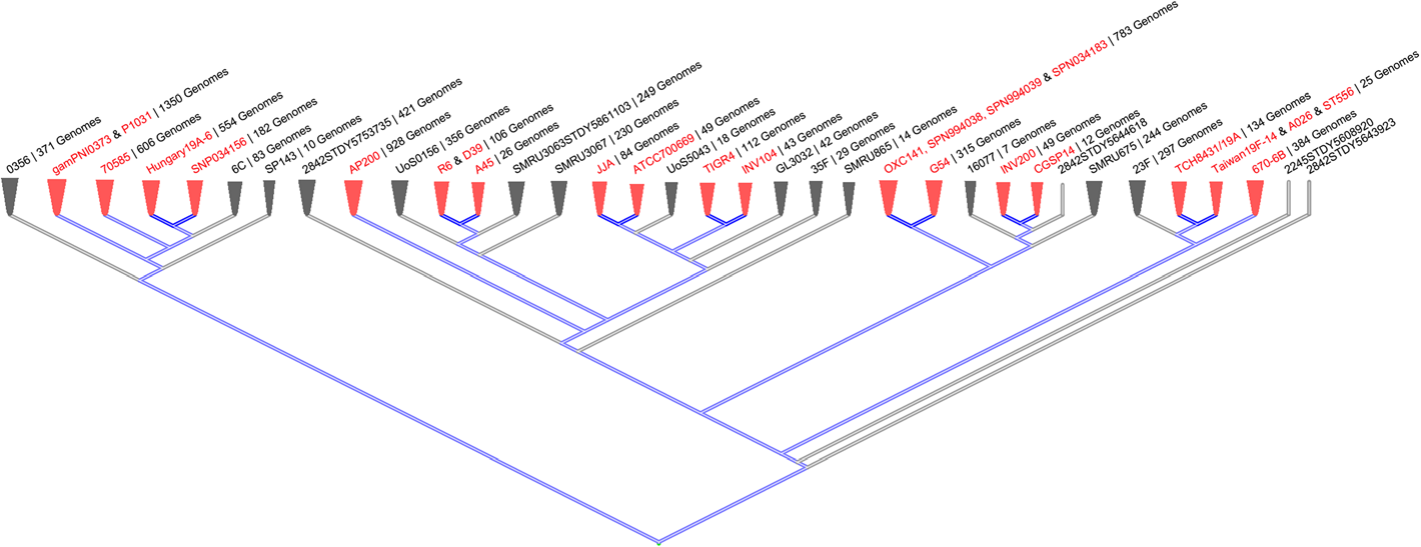
Phylogenetic tree (slanted cladogram) of the pneumococcal genome / strains. By using the online NCBI Tools *Genome Tree Report* (ncbi.nlm.nih.gov/genome/tree/176) and the *Tree Viewer 1.17.0* (ncbi.nlm.nih.gov/projects/treeview), a phylogenetic tree was constructed from the analysis by genomic BLAST of 8290 sequencing projects of pneumococci reported in the NCBI database. The topology of this slanted cladogram showed different pneumococcal lineages, where the selected set of 25 pneumococcal strains can be identified in red as external nodes (the “well-distributed” key features also highlighted in red), evidencing an optimal representation of the pneumococcal population. The overall number of sequenced pneumococcal genomes is provided for each external node. The blue lines depicted those external nodes where fully sequenced and annotated genomes are located

The search and selection of the *Streptococcus pneumoniae* strains for the analysis in this study was done using the microbial database of the “*National Center for Biotechnology Information*” NCBI (http://www.ncbi.nlm.nih.gov/genome) [22]. Likewise, in order to ensure an optimal representation of the global pneumococcal population, a genomic BLAST of 8290 available *S. pneumoniae* genomes was carried out. In brief, DNA alignments, employing the tool “*Microbial Nucleotide BLAST*” [23], that can be found in the website http://blast.ncbi.nlm.nih.gov/Blast.cgi, were performed for all the currently reported draft or complete sequenced genomes. The comparative data was then employed to construct a DNA-based Phylogenetic Tree (dendrogram), by using the Genome Tree Report Tool of the NCBI (ncbi.nlm.nih.gov/genome/tree/176). Afterwards, the file containing the dendrogram, constructed for the 8290 strains, was downloaded from the NCBI database. Finally, the dendrogram file was viewed, analyzed and adapted in order to generate circular, slanted and/or rectangular cladograms, by using the online NCBI Tool “Tree Viewer 1.17.0”, which is available online at the website: ncbi.nlm.nih.gov/projects/treeview (Fig. 1).

### Definition of the virulence factors and two-component regulatory systems to be studied in *S. pneumoniae*

The search and selection of genes and proteins widely known as virulence factors or gene encoding factors possessing a proven interaction with the human host was done by an exhaustive bioinformatic screening in the database “*Virulence Factors DataBase - VFDB*” [24], available at the website http://www.mgc.ac.cn/VFs. Aditionally, the virulence factors and proteins involved in interactions with the host were confirmed and completed by a systematic review of the literature [14, 25]. The common names of each one of the selected virulence factors were then introduced in the database UNIPROT [26], available at http://www.uniprot.org/, with the aim of obtaining the locus tag for *S. pneumoniae* TIGR4 genome/strain. In addition, the genes encoding the HK or RR of the pneumococcal TCS were identified by using the database Prokaryotic Two-Component Systems - P2CS [27], available at the website http://www.p2cs.org/index.php. Likewise, the corresponding locus tag for *S. pneumoniae* TIGR4 genome / strain, of each one of the histidine kinases genes (*hk*) and response regulator genes (*rr*), were also recovered from the same database.

### Chromosomal localization of the virulence factor and two-component regulatory systems genes in *S. pneumoniae*

The chromosomal location of all the genes in the genome of *S. pneumoniae* TIGR4 and the construction of the genomic maps, in linear or circular representation, was done by using the software SnapGene® (GSL Biotech), available at http://www.snapgene.com. In brief, the studied genomes of *S. pneumoniae* were imported through its corresponding access code in GenBank (ie: NC_003028.3 for TIGR4). Then, the chromosomal location of each virulence factor gene, and the factors involved in the interaction with the host and the genes encodying for proteins of simple or two-component regulatory systems were identified. Finally, the lineal maps for the scale genomic localization for the virulence factors and the circular maps for the genomic periphery of the genes that form the two-component regulatory systems were constructed.

### Distribution of the virulence factors and two-component regulatory systems in the different strains of *S. pneumoniae*

The identification of the genetic and protein sequences of interest to perform the comparative analysis was done, having as reference the codes (Locus Tag) in the genomes of *S. pneumoniae* TIGR4 and/or R6 in the database Kyoto Encyclopedia of Genes and Genomes – KEGG [28], available at http://www.kegg.jp/kegg/. Once every gene of interest was established in the database, a series of comparisons (BLASTs) were performed using the GenomeNet [29], available at http://www.genome.jp/, using only the fully sequenced and annotated genomes of *S. pneumoniae*. For the nucleotide sequences the search was performed using the program BLASTN 2.2.29+, which uses nucleotide vs nucleotide alignments based on a punctuation matrix BLOSUM62 [23, 30]. In the same way, the search was done for the amino acid sequences using the program BLASTP 2.2.29+ [31, 32], that performs amino acids vs amino acids alignments based on a similar matrix. Once the BLAST was finalized for each virulence factor, the list was purged using as selection criteria genes with an expectancy value: e-Value = 0. The inclusion of genes with an e-value >0 was done by direct visual inspection of the alignments to check that it was indeed the same sequence. By having defined the list with the genes and proteins that fulfilled the selection criteria, it was defined to which strains of *S. pneumoniae* they belong. All the DNA and protein sequences were downloaded and stored in an organized way using the fasta format.

### Genetic variability (variome) of the virulence factors and two-component regulatory systems among the different pneumocococal strains

The multiple comparative alignments of pneumococcal sequences were done using the web tool MultAlin [33], available at http://multalin.toulouse.inra.fr/multalin/, for which an identity matrix 1–0 was used to assign a penalty even for the slightest change in the nucleotides or amino acids sequences, covering substitution, deletions, insertions and variations in the length. From these analyses, the number of allelic and protein variants were determined for each gene according to the registry value assigned by the program to each sequence, where equal sequences have the same registry value, while different sequences possess different values. The results of the alignments were manually curated and stored for further analysis. Finally, the precise determination of the total mutations, synonymous and nonsynonymous was done using the software DnaSP V.5.1 [34, 35], available at http://www.ub.edu/dnasp/. There, all the sequences found for a determined gene were introduced and the calculations were perfomed for the corresponding type of mutation as mentioned before.

## Results and discussion

“Hundreds to thousands” of *S. pneumoniae* strains and clinical isolates recovered from the nasopharynx, blood or cerebrospinal fluid (CSF) have been included up to date in genomic sequencing projects worldwide. However, pneumococcal strains, whose genomes are fully sequenced, annotated and publicly available, are the focus of this study. Therefore, a set of 25 pneumococcal strains were selected from the NCBI database, as population study, to perform the bioinformatic analysis needed to accomplish the construction of the variome of the virulence factors and two-component regulatory systems of *Streptococcus pneumoniae* (Table 1).

**Table 1:** The study population set of 25 *S. pneumoniae* strains included in this study and their serotypes

*S. pneumoniae* Strain	Serotype	# of Genes	NCBI Annotation
D39	2	2069	NC_008533.1
R6	No Capsule	1967	NC_003098.1
TIGR4	4	2228	NC_003028.3
INV104	1	2003	NC_017591.1
AP200	11A	2284	NC_014494.1
JJA	14	2235	NC_012466.1
ATCC 700669	23F	2224	NC_011900.1
INV200	14	2113	NC_017593.1
CGSP14	14	2276	NC_010582.1
G54	19F	2186	NC_011072.1
gamPNI0373	1	2226	NC_018630.1
P1031	1	2254	NC_012467.1
SPN034156	3	1956	NC_021006.1
SPN994039	3	1974	NC_021005.1
SPN994038	3	1974	NC_021026.1
SPN034183	3	1985	NC_021028.1
OXC141	3	2037	NC_017592.1
670-6B	6B	2430	NC_014498.1
A026	19F	2153	NC_022655.1
Taiwan19F-14	19F	2205	NC_012469.1
ST556	19F	2219	NC_017769.1
TCH8431/19A	19A	2355	NC_014251.1
SPNA45	3	1921	NC_018594.1
70,585	5	2323	NC_012468.1
Hungary19A 6	19A	2402	NC_010380.1

A Variome model of the Pneumococcal Virulence Factors and Regulators is an intraspecific study, aiming to highlight variable genetic loci on the genome of *Streptococcus pneumonie*. A perfect and ultimate Variome model would be that constructed with the 100% of the genomic information correctly assessed from the entire pneumococcal population. However, the current state of the art is far away from this scenario and an optimal representation of the pneumococcal sets assessed up to date would be appropriate in order to validate these genomic analyzes. Currently, 8290 pneumococcal sequencing projects are reported as draft or complete genomes in the Genome Assembly and Annotation Report of the NCBI database. Therefore, a global genomic BLAST (DNA alignment) of those 8290 available *S. pneumoniae* genomes/strains was performed and a DNA-based Phylogenetic Tree was constructed by using the Genome Tree Report Tool of the NCBI. The topology of this phylogenetic tree (slanted cladogram) showed different pneumococcal lineages, where the selected set of 25 pneumococcal genomes/strains can be identified as external nodes (“well-distributed” key features highlighted in red), evidencing an optimal representation of the pneumococcal population (Fig. 1). In addition, it is important to highlight that the serotypes (1, 2, 3, 4, 5, 6B, 11A, 14, 19A, 19F and 23F), represented in this study population set, have been described as the pneumococcal types with the highest pathogenic potencial, due to the high burden of invasive pneumococcal diseases (IPDs) they cause worldwide. This is the reason why the majority of them (except serotypes 2 and 11A) have been included in the pneumococcal conjugate vaccines (PCVs) currently used for immunization [1].

An initial considerable number of pneumococcal virulence factor genes were identified, by employing the database *VFDB *[24]. This database provided further detailed information to establish their function, pathogenic role and type of interaction with a receptor in its human host. Aditionally, a systematic screening of the literature [14] did not only allow the confirmation of identified factors, but also ensured the posibility to complement the list with additional factors that have not been included in the databases. Likewise, the number of the *tcs* genes (27) was determined using the database Prokaryotic 2-Component Systems - P2CS [27]. In total, 92 different genes encoding 61 surface proteins, 4 stand alone transcriptional regulators, 13 HKs and 14 RRs have been selected and included in this work for the construction of the variome, after being classified by their function and grouped according to their molecular mechanisms of surface-exposure (Table 2).

**Table 2:** Function or pathogenic role of the virulence factors and two-component regulatory systems of *S. pneumoniae*

Virulence Factors		Protein Name	Function and/or Pathogenic Role
LPxTG - Proteins	BgaA	β-Galactosidase	β-Galactosidase Enzyme
	EndoD	Endo-β-N-Acetylglucosaminidase D	Virulence
	PclA	Pneumococcal Collagen-Like Protein	Adherence and Invasion
	SpGH101	Endo-α-N-Acetylgalactosaminidase	Virulence
	StrH	β-N-Acetylhexosaminidase	β-N-Acetylhexosaminidase Enzyme
	NanA	Neuraminidase A	Hydrolytic Enzyme, Adherence and Colonization
	PfbA	Plasmin- and Fibronectin-Binding Protein A	Adherence, Immune Evasion and Antiphagocytosis
	PrtA	Subtilysin-Like Serine Protease	Virulence
	PavB	Pneumococcal Adherence and Virulence Protein B	Adherence and Colonization
	KsgA	Dimethyladenosine Transferase	Virulence
	SpuA	Alkaline Amyllopullullanase	Pullullanase Enzyme and Immune Evasion
	HysA	Hyaluronate Lyase	Hyaluronidase Enzyme and Colonization
	SP_1492	Cell Wall Surface Anchor Protein Family, Mucin-Binding Protein	Virulence
	ZmpA	Zinc Metalloprotease A, IgA1	IgA1 Protease Enzyme and Colonization
	ZmpB	Zinc Metalloprotease B	Immune Evasion and Colonization
	ZmpC	Zinc Metalloprotease C	Immune Evasion and Colonization
	ZmpD	Zinc Metalloprotease D, IgA1 Paralog Protease	Immune Evasion and Colonization
	PsrP	Pneumococcal Serine-Rich Repeats Protein	Adherence
	RrgA	Pilus-1 Tip Protein (Adhesin)	Adherence
	RrgB	Pilus-1 Backbone Protein	Adherence
	RrgC	Pilus-1 Anchore Protein	Adherence
	PitA	Pilus-2 Subunit, Ancillary Protein	Adherence
	PitB	Pilus-2 Subunit, Backbone Protein	Adherence
Choline-Binding Proteins (CBPs)	LytA	Autolysin (N-Acetyl-Muramoyl-L-Alanine Amidase)	Autolytic Enzyme, Cell Wall Digestion and Autolysis
	LytB	Endo-β-N-Acetylglucosamidase	Immune Evasion and Colonization
	LytC	Lysozyme (1,4-β-N-Acetylmuramidase)	Adherence, Immune Evasion and Colonization
	Pce	Choline-Binding Protein E, Phosphorylcholine Estearase	Phosphorylcholine Estearase Enzyme, Adherence, Colonization and Cellular Metabolism
	PcpA	Pneumococcal Choline-Binding Protein A	Protection Against Lung Infection and Sepsis
	PspA	Pneumococcal Surface Protein A	Cellular Metabolism and Immune Evasion
	PspC	Pneumococcal Surface Protein C, Choline-Binding Protein A	Adherence, Immune Evasion, Colonization and Invasion
	CbpC	Choline-Binding Protein C	Virulence
	CbpD	Choline-Binding Protein D	Colonization
	CbpF	Choline-Binding Protein F	Virulence
	CbpG	Choline-Binding Protein G	Adherence and Colonization
	CbpI	Choline-Binding Protein I	Virulence
	CbpJ	Choline-Binding Protein J	Virulence
	SP_0667	Pneumococcal Surface Protein (Putative Lysozyme)	Virulence
Lipoproteins	GlnQ	Glutamine Transporter	Cellular Metabolism
	PiaA	Iron-Compound ABC Transporter	Peptidil-Prolil Isomerase (PPIase) Enzyme
	PiuA	Iron-Compound ABC Transporter	Peptidil-Prolil Isomerase (PPIase) Enzyme
	PpiA	Streptococcal Lipoprotein Rotamase A, Peptidil-Prolil Isomerase (PPIases) Enzyme	Peptidil-Prolil Isomerase (PPIase) Enzyme
	PsaA	Peptide Permease Enzyme, Manganese ABC Transporter, Manganese-Binding Lipoprotein	Immune Evasion
	PpmA	Foldase Protein PrsA, Proteinase Maturation A	Adherence, Immune Evasion, Strain-Specific Colonization and Evasion of Phagocytosis
	AliA	Oligopeptide ABC Trasporter	Adherence
	PhtA	Pneumococcal Histidine Triad A	Adherence and Immune Evasion
	PhtB	Pneumococcal Histidine Triad B	Adherence and Immune Evasion
	PhtD	Pneumococcal Histidine Triad D	Adherence and Immune Evasion
	PhtE	Pneumococcal Histidine Triad E	Adherence and Immune Evasion
Non-Classical Surface-Exposed Proteins	Eno	Enolase (2-Phosphoglycerate Dehydratase)	Glycolytic Enzyme, Adherence and Colonization
	GAPDH	Glyceraldehyde-3-Phosphate Dehydrogenase	Glycolytic Enzyme, Adherence and Colonization
	HtrA	High-Temperature Requirement A, Serine Protease (Heat Shock Protein)	Serine Protease Enzyme
	PavA	Pneumococcal Adherence and Virulence Protein A	Adherence, Immune Evasion, Colonization and Translocation
	Pbp1B	Penicillin-Binding Protein 1B	Antibiotic Resistance
	StkP	Serine/Threonine Protein Kinase	Cellular Metabolism and Fitness
	Usp45	PcsB, Secreted 45-KDa Protein	Virulence
	NanB	Neuraminidase B	Hydrolytic Enzyme, Adherence and Colonization
	PppA	Pneumococcal Protective Protein A, Non-Heme Iron-Containing Ferritine	Colonization
	Fic-Like	Fic-Like Cell Fillamentation Protein	Putative Cytotoxicity
	6PGD	6-Phosphogluconate Dehydrogenase	Virulence
	Ply	Pneumolysin	Cytolytic Toxin, Adherence, Immune Evasion, Invasion, Dissemination and Complement Activation
	NanC	Neuraminidase C	Virulence
Regulators	RlrA	Pathogenicity Island *rlrA* Transcriptional Regulator	Virulence
	MgrA	MgrA Family Transcriptional Regulator	Virulence
	MerR	MerR Family Transcriptional Regulator	Virulence
	PsaR	Iron-Dependent Transcriptional Regulator	Virulence
Histidine Kinases (HKs)	HK01	Sensor Histidine Kinase	Virulence
	HK02	Sensor Histidine Kinase, VicK	Antibiotic Resistance, Virulence and Fitness
	HK03	Sensor Histidine Kinase, LiaS	Antibiotic Resistance and Stress Protection
	HK04	Sensor Histidine Kinase, PnpS	Genetic Competence, Fitness, Immune Evasion
	HK05	Sensor Histidine Kinase, CiaH	Antibiotic Resistance, Genetic Competence and Pathogenesis
	HK06	Sensor Histidine Kinase	Colonization and Invasion
	HK07	Sensor Histidine Kinase, YesM	Fitness
	HK08	Sensor Histidine Kinase, SaeS	Pathogenesis and Fitness
	HK09	Sensor Histidine Kinase	Virulence
	HK10	Sensor Histidine Kinase, VncS	Antibiotic Resistance
	HK11	Sensor Histidine Kinase	Biofilm Formation
	HK12	Sensor Histidine Kinase, ComD	Genetic Competence
	HK13	Sensor Histidine Kinase, BlpH	Virulence
Response Regulators (RRs)	RR01	Response Regulator	Virulence
	RR02	Response Regulator, VicR	Antibiotic Resistance, Virulence and Fitness
	RR03	Response Regulator, LiaR	Antibiotic Resistance and Stress Protection
	RR04	Response Regulator, PnpR	Genetic Competence, Fitness, Immune Evasion
	RR05	Response Regulator, CiaR	Antibiotic Resistance, Genetic Competence, Pathogenesis
	RR06	Response Regulator	Colonization and Invasion
	RR07	Response Regulator, YesN	Fitness
	RR08	Response Regulator, SaeR	Pathogenesis and Fitness
	RR09	Response Regulator	Virulence
	RR10	Response Regulator, VncR	Antibiotic Resistance
	RR11	Response Regulator	Biofilm Formation
	RR12	Response Regulator, ComE	Genetic Competence
	RR13	Response Regulator, BlpR	Virulence
	RR14	Response Regulator	Virulence

The proteins are grouped by classes, depending on their surface-exposure mechanism. The names, abreviations and function of the proteins were obtained from literature references

The genomes of 25 analyzed pneumococcal strains comprise genome sizes ranging from 2,024,476 bp in SPN034156 up to 2,245,615 bp in Hungary 19A-6. Likewise, the G + C content varies between 39.50% in CGSP14 and 39.90% in SPN034156. 670-6B is the strain with the highest number of genes (2430) and proteins (2352) and SPN034156 is the strain with the lowest number of genes (1956) and proteins (1799). Hence, the difference among genomes, regarding the number of genes and proteins can be up to 474 genes and 553 proteins, respectively. The overall number of genes for each pneumococcal genome evaluated here overmatches the overall number of proteins because the reported number of genes includes all the tRNA-, rRNA- and protein-encoding genes.

**Fig. 2:**
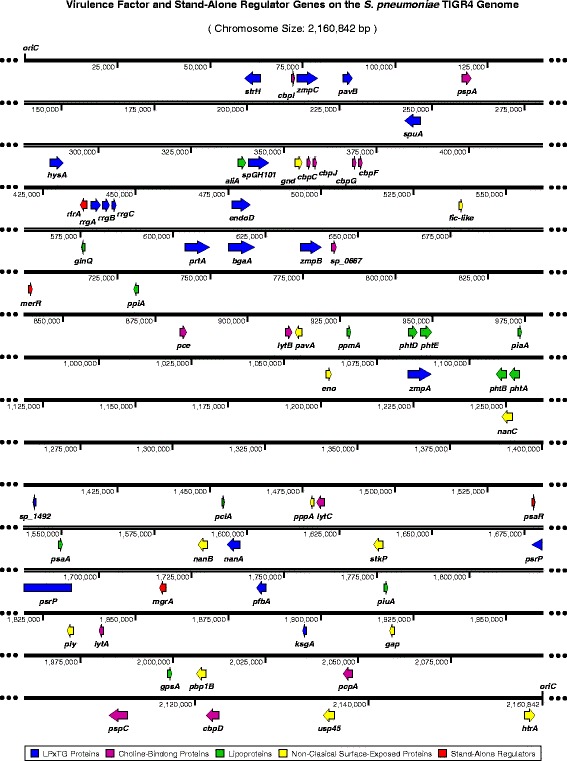
Chromosomal localization and direction of the virulence factor genes of *S. pneumoniae* TIGR4. Lineal representation of the pneumococcus genome. The arrows, drawn at scale, localize 62 of the 65 virulence factors and simple regulation genes considered in this study (*pitA*, *pitB* and *zmpD* are not present in the genome of TIGR4). Each color represents a different class of codified protein: blue = sortase-anchored proteins with an LPxTG cleavage motif; violet = choline-binding proteins (CBPs); green = lipoproteins, yellow = non-classical surface proteins (NCSP), and red = stand-alone regulators. This map was constructed using the Software SnapGene® (GSL Biotech; Available at snapgene.com)

**Fig. 3:**
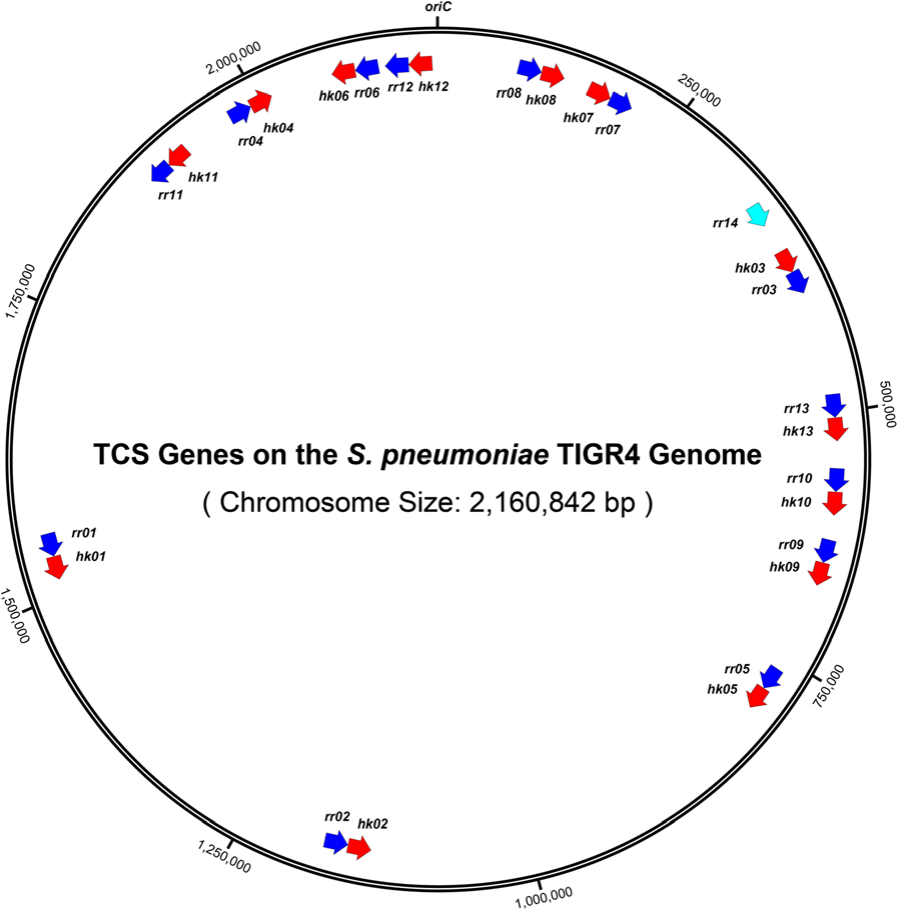
Localization and direction of the two component systems genes in *S. pneumoniae* TIGR4. Circular representation of the pneumococcal genome. The arrows, not drawn at scale, localize the 27 genes which codifies for the proteins of the 13 two component systems +1 incomplete. Each color indicates a different class of codified protein: red = histidine kinase sensors and blue = response regulators Proteins. This map was constructed using the Software SnapGene® (GSL Biotech; Available at snapgene.com)

Considering the chromosomal localization of pneumococcal virulence factors genes, they are all distributed along the pneumococcal genome (Fig. 2). Interestingly, these genes are located in a co-oriented manner in relation with the origin of replication (*oriC*: 2.160.822–196). During the bidirectional replication of the genome, gene transcription must be simultaneous [36]. Hence, for the genes oriented in opposite direction to the corresponding replication fork, both molecular machineries will run into a frontal collision that might affect at least one of the processes. For replication, this phenomenon implies a genomic instability, while the gene transcription is probably inefficient. Previous studies have proven that the essential and highly constitutively expressed genes are co-oriented [36]. For the pneumococcus, 30 of the 36 genes encoding virulence factors are localized in the first half of the genome, on the forward strand, and co-oriented with the replication fork clockwise. Similarly, 21 of the 27 virulence factor genes localized on the second half of the genome, are located on the reverse strand and co-oriented with the replication fork moving anti-clockwise (Fig. 2). A similar genome organization is observed for the 27 genes that encode the TCSs in *S. pneumoniae*, where only one operon, the *tcs*04 genes (TCS04), is not co-oriented with the replication fork (Fig. 3). These data reinforce the idea that the virulence factor genes and the genes of the *tcs* are highly important for the pneumococcal interaction with the human host, and its pathogenic potential in processes such as adherence, colonization, invasion, immune evasion, fitness, antibiotic resistance and natural competence (Table 2).

The analysis of the distribution of genes associated with virulence and host-pathogen interactions among the studied pneumococcal strains revealed that only 26 of the 65 genes considered here are present in the all 25 strains. These genes encode for products involved in different functions such as cell wall hydrolysis, ABC transporters and structural proteins implied in the adherence to host tissue, the so-called adhesins. Interestingly, after preliminar inspection (by *locus tag*, *identifier names* and/or *product sizes*) of the datasets and supplementary material reported by van Tonder and colleagues in 2017, only a few of the pneumococcal virulence factors (PspC, KsgA, and 4 hypothetical lipoproteins) and regulators (RR04, HK08, RR08, RR09, RR10) were found in the pneumococcal “*supercore*” genomic list of 303 genes, based on the analysis of 3121 pneumococci recovered from healthy individuals from four different subsets of the global pneumococcal population [15]. These findings, if confirmed after deeper analysis of the datasets based on sequence comparison, may indicate that pneumococcal pathogenesis is a much more complex process than thought before. While most of the genes have a single copy in the genome, the *lytA* gene, encoding the major pneumococcal autolysin, is found also in two and even three copies in 13, and 2 strains, respectively. This is most likely due to the multiple integration of prophages in the chromosomal DNA [37] (Table 3). In strain SPNA45, the gene *gnd*, encoding the enzyme 6-phophogluconate dehydrogenase, is duplicated and fused with a second copy of its downstream neighbor gene, which encodes the orphan response regulator (*rr14*). The remaining 39 of the 65 virulence factor genes were found to belong to the accesory genome, presenting different degrees of absence in the 25 strains. Thus, all these genes are not essential but are beneficial for fitness and pathogenesis. Striking examples are the genes encoding the Pilus-1 and Pilus-2 structures that have been identified to mediate adherence, contribute to virulence and promote invasion [38–42]. These genes are located on pathogenicity islands (PAI) and these islands contain also the genes required for cell surface anchoring and regulation [38–41]. Remarkably, strains like ST556, Taiwan19F-14 and TCH8431/19A, were detected here as positive for both types of pili (1 and 2). Among the other genes with restricted presence in some strains it is important to mention that they encode for sortase-anchored proteins or choline-binding proteins (CBPs), as well as histidine triad proteins (*pht* genes). These gene products are associated with different processes of bacterial fitness and pathogenesis (Tables 3 and 2) [6, 43, 44]. Regarding the distribution and data of the analyzed strains for the TCS most of them were found in the 25 pneumococcal strains. Exceptions are presented by the TCS07 and TCS12, which contribute to fitness and competence, respectively [7, 45]. These TCS are absent in a couple of strains (Table 4). In some other strains genes like *hk01*, *hk12* and *rr04*, presented incomplete sequences, an artefact leading to truncated and hence non-functional proteins/regulators (Tables 3 and 2). Interestingly, only the genes encoding the *hk08*, *rr08*, *rr09*, *rr06* and *rr04* were found to belong to the “*supercore*” genomic set of genes reported by van Tonder et al., in 2017 [15], indicating the important role these highly conserved and well-distributed regulatory proteins play in the pneumococcus and in its interplay with the environment.

**Table 3:** Distribution of the virulence factor and regulation genes of *S. pneumoniae*

Virulence Factors		TIGR4	D39	R6	70,585	Hungary19A 6	SPN994038	SPN994039	SPN034183	OXC141	SPN034156	ST556	Taiwan19F-14	TCH8431/19A	A026	INV200	CGSP14	INV104	G54	AP200	P1031	gamPNI0373	SPNA45	ATCC 700669	JJA	670-6B
LPxTG - Proteins	*bgaA*	1	1	1	1	1	1	1	1	1	1	1	1	1	1	1	1	1	1	1	1	1	1	1	1	1
	*endoD*	1	1	1	1	1	1	1	1	1	1	1	1	1	1	1	1	1	1	1	1	1	1	1	1	1
	*pclA*	1	1	1	1	1	1	1	1	1	1	1	1	1	1	1	1	1	1	1	1	1	1	1	1	1
	*spGH101*	1	1	1	1	1	1	1	1	1	1	1	1	1	1	1	1	1	1	1	1	1	1	1	1	1
	*strH*	1	1	1	1	1	1	1	1	1	1	1	1	1	1	1	1	1	1	1	1	1	1	1	1	1
	*nanA*	**1**	1	1	1	1	1	1	1	1	1	1	1	1	1	1	1	1	1	1	1	1	1	1	1	1
	*pfbA*	1	1	1	1	1	1	1	1	1	1	1	1	1	1	1	1	1	1	1	1	1	0	1	1	1
	*prtA*	1	1	1	1	1	1	1	1	1	1	1	1	1	1	1	1	1	1	1	1	1	0	1	1	1
	*pavB*	1	1	1	1	1	1	1	1	1	1	1	1	1	1	1	1	1	1	1	1	0	1	0	1	1
	*ksgA*	1	1	1	1	1	1	1	1	0	1	1	1	1	1	1	1	1	1	1	1	0	1	0	1	1
	*spuA*	1	1	1	1	1	1	1	1	1	0	1	1	1	1	1	1	1	1	1	1	1	1	1	1	1
	*hysA*	1	1	1	1	1	0	0	0	0	0	1	1	1	1	1	1	1	1	1	1	1	1	1	1	1
	*SP_1492*	1	1	1	1	1	0	0	0	0	0	1	1	1	1	1	0	1	1	1	1	1	1	0	1	1
	*zmpA*	1	1	1	1	1	1	1	1	1	1	1	1	1	1	0	0	1	0	1	0	0	0	0	0	1
	*zmpB*	1	1	1	1	1	1	1	1	1	1	1	1	1	1	1	1	1	1	1	1	1	0	1	0	1
	*zmpC*	1	0	0	0	0	0	0	0	0	0	0	0	0	0	0	0	0	1	1	0	0	0	0	0	0
	*zmpD*	0	0	0	0	0	0	0	0	0	0	0	0	0	0	1	1	0	1	0	1	1	0	1	1	0
	*psrP*	1	0	0	1	1	0	0	0	0	0	0	0	0	0	0	1	1	0	0	0	0	0	0	0	0
	*rrgA*	1	0	0	0	1	0	0	0	0	0	1	1	1	1	0	0	0	0	0	0	0	0	0	0	1
	*rrgB*	1	0	0	0	1	0	0	0	0	0	1	1	1	1	0	0	0	0	0	0	0	0	0	0	1
	*rrgC*	1	0	0	0	1	0	0	0	0	0	1	1	1	1	0	0	0	0	0	0	0	0	0	0	1
	*pitA*	0	0	0	0	0	1	0	0	0	0	1	1	1	0	0	0	1	0	1	0	0	0	0	0	0
	*pitB*	0	0	0	0	0	1	0	0	0	0	1	1	1	0	0	0	1	0	1	0	0	0	0	0	0
Choline-Binding Proteins (CBPs)	*lytA*	1	1	1	**2**	**2**	**2**	**2**	**2**	**2**	**2**	**2**	1	1	1	1	1	1	1	**2**	**2**	**2**	***2***	**2**	**3**	**3**
	*lytB*	1	1	1	1	1	1	1	1	1	1	1	1	1	1	1	1	1	1	1	1	1	1	1	1	1
	*lytC*	1	1	1	1	1	1	0	0	0	0	1	1	1	0	1	1	1	1	1	1	1	1	1	1	1
	*pce*	1	1	1	1	1	1	1	1	1	1	1	1	1	1	1	1	1	1	1	1	1	1	1	1	1
	*pcpA*	1	1	1	1	1	0	0	0	0	0	1	1	1	1	1	1	1	1	1	0	0	1	1	1	1
	*pspA*	1	1	1	1	1	1	1	1	1	1	1	1	1	1	1	1	1	1	1	1	1	0	***1***	0	1
	*pspC*	1	1	1	1	1	0	0	0	0	1	1	1	1	1	0	1	1	0	1	1	1	1	1	1	1
	*cbpC*	1	1	1	1	1	1	1	1	1	1	1	1	1	1	1	0	1	1	1	1	1	0	0	1	1
	*cbpD*	1	1	1	1	1	1	1	1	1	1	1	1	1	1	1	1	1	1	1	1	1	1	1	1	1
	*cbpF*	1	1	1	1	1	0	0	0	0	0	0	0	0	0	0	0	0	0	0	0	0	0	1	1	1
	*cbpG*	1	0	0	0	0	0	0	0	1	1	1	1	1	1	1	1	1	1	1	1	1	1	1	1	0
	*cbpI*	1	0	0	0	0	0	0	0	0	0	0	0	0	0	0	0	0	1	1	0	0	0	0	0	0
	*cbpJ*	1	0	0	0	0	1	1	1	1	1	1	1	1	1	1	1	0	0	0	0	0	0	1	1	1
	*SP_0667*	1	1	1	1	1	1	1	1	1	1	1	1	1	1	1	1	1	1	1	0	0	0	0	0	0
Lipoproteins	*glnQ*	1	1	1	1	1	1	1	1	1	1	1	1	1	1	1	1	1	1	1	1	1	1	1	1	1
	*piaA*	1	1	1	1	1	1	1	1	1	1	1	1	1	1	1	1	1	1	1	1	1	1	1	1	1
	*piuA*	1	1	1	1	1	1	1	1	1	1	1	1	1	1	1	1	1	1	1	1	1	1	1	1	1
	*ppiA*	1	1	1	1	1	1	1	1	1	1	1	1	1	1	1	1	1	1	1	1	1	1	1	1	1
	*psaA*	1	1	1	1	1	1	1	1	1	1	1	1	1	1	1	1	1	1	1	1	1	1	1	1	1
	*ppmA*	1	1	1	1	1	1	1	1	1	1	1	1	1	1	0	1	1	0	0	1	1	1	1	1	1
	*aliA*	1	1	1	1	1	0	0	0	0	0	1	1	1	1	1	1	1	1	1	1	1	0	1	1	1
	*phtA*	1	1	1	1	0	0	0	0	0	0	0	0	0	0	0	0	1	0	1	1	1	0	0	0	1
	*phtB*	1	0	0	0	0	0	0	0	0	0	1	1	0	0	1	0	1	1	1	0	1	0	0	0	0
	*phtD*	1	1	1	1	1	1	1	1	1	0	1	1	1	0	1	1	1	1	1	1	1	1	1	1	1
	*phtE*	1	1	1	1	1	1	1	1	1	1	1	1	1	1	1	1	1	1	1	1	1	1	1	1	1
Non-Classical Surface-Exposed Proteins	*eno*	1	1	1	1	1	1	1	1	1	1	1	1	1	1	1	1	1	1	1	1	1	1	1	1	1
	*gap*	1	1	1	1	1	1	1	1	1	1	1	1	1	1	1	1	1	1	1	1	1	1	1	1	1
	*htrA*	1	1	1	1	1	1	1	1	1	1	1	1	1	1	1	1	1	1	1	1	1	1	1	1	1
	*pavA*	1	1	1	1	1	1	1	1	1	1	1	1	1	1	1	1	1	1	1	1	1	1	1	1	1
	*pbp1B*	1	1	1	1	1	1	1	1	1	1	1	1	1	1	1	1	1	1	1	1	1	1	1	1	1
	*stkP*	1	1	1	1	1	1	1	1	1	1	1	1	1	1	1	1	1	1	1	1	1	1	1	1	1
	*usp45*	1	1	1	1	1	1	1	1	1	1	1	1	1	1	1	1	1	1	1	1	1	1	1	1	1
	*nanB*	1	1	1	1	0	1	1	1	1	1	1	1	1	1	1	1	1	1	1	1	1	1	1	1	1
	*pppA*	1	1	1	1	1	1	1	0	1	1	1	1	1	1	1	1	1	1	1	1	1	1	1	1	1
	*fic*-Like	1	1	1	1	1	1	1	1	1	1	1	1	1	1	1	1	1	1	0	1	1	1	1	1	1
	*gnd*	1	1	1	1	1	1	1	1	1	1	1	1	1	1	1	1	1	1	1	1	1	**2**	1	1	1
	*ply*	1	1	1	1	1	1	1	1	1	1	1	1	1	1	1	1	1	1	1	1	1	***1***	1	1	1
	*nanC*	1	0	0	0	1	0	0	0	0	0	0	0	0	0	1	1	1	1	1	0	0	0	1	1	1
Regulators	*mgrA*	1	1	1	1	1	1	1	1	1	1	1	1	1	1	1	1	1	1	1	1	1	1	1	1	1
	*psaR*	1	1	1	1	1	1	1	1	1	1	1	1	1	1	1	1	1	1	1	1	1	1	1	1	1
	*merR*	1	0	1	0	0	1	1	1	1	1	1	0	1	1	1	1	0	1	1	1	1	0	1	1	1
	*rlrA*	1	0	0	0	1	0	0	0	0	0	1	1	1	1	0	0	0	0	0	0	0	0	0	0	1

The present table shows the absence (0) or presence (1, 2 or 3) of each considered genes in the 25 strains selected for this study. The number (1, 2 or 3) indicates the amount of copies of each gene in the genome. *lytA* is the only factor with more than one copy per genome. In the strain SPNA45, the gene *gnd* was found duplicated (**2**) and fused with a duplication of its neighbor gene (*rr14*) downstream. In the gene *nanA* of TIGR4 (**1**) a shift in its ORF was found. However, it has also been reported that NanA is expressed in this pneumoccoccal strain. Gene defective copies (genes with any alteration in their primary DNA sequences) are depicted in bold and italics: In the SPNA45 strain *ply* is fused with a copy of *lytA*, and *pspA* is defective in the ATCC700669 pneumococcal strain

**Table 4:** Distribution of the genes that conform the two-component systems in *S. pneumoniae*

Two-Component Systems (TCSs)		TIGR4	D39	R6	70,585	Hungary19A 6	SPN994038	SPN994039	SPN034183	OXC141	SPN034156	ST556	Taiwan19F-14	TCH8431/19A	A026	INV200	CGSP14	INV104	G54	AP200	P1031	gamPNI0373	SPNA45	ATCC 700669	JJA	670-6B
Histidine Kinases (HKs)	*hk01*	1	1	1	1	1	1	1	1	1	1	1	1	1	1	1	1	1	1	***1***	1	1	1	1	1	1
	*hk02*	1	1	1	1	1	1	1	1	1	1	1	1	1	1	1	1	1	1	1	1	1	1	1	1	1
	*hk03*	1	1	1	1	1	1	1	1	1	1	1	1	1	1	1	1	1	1	1	1	1	1	1	1	1
	*hk04*	1	1	1	1	1	1	1	1	1	1	1	1	1	1	1	1	1	1	1	1	1	1	1	1	1
	*hk05*	1	1	1	1	1	1	1	1	1	1	1	1	1	1	1	1	1	1	1	1	1	1	1	1	1
	*hk06*	1	1	1	1	1	1	1	1	1	1	1	1	1	1	1	1	1	1	1	1	1	1	1	1	1
	*hk07*	1	1	1	1	1	1	1	1	1	1	1	1	1	1	1	0	1	1	1	1	1	1	1	1	1
	*hk08*	1	1	1	1	1	1	1	1	1	1	1	1	1	1	1	1	1	1	1	1	1	1	1	1	1
	*hk09*	1	1	1	1	1	1	1	1	1	1	1	1	1	1	1	1	1	1	1	1	1	1	1	1	1
	*hk10*	1	1	1	1	1	1	1	1	1	1	1	1	1	1	1	1	1	1	1	1	1	1	1	1	1
	*hk11*	1	1	1	1	1	1	1	1	1	1	1	1	1	1	1	1	1	1	1	1	1	1	1	1	1
	*hk12*	1	1	1	1	1	1	1	1	1	1	***1***	1	1	1	1	1	0	1	1	1	1	0	1	1	1
	*hk13*	1	1	1	1	1	1	1	1	1	1	1	1	1	1	1	1	1	1	1	1	1	1	1	1	1
Response Regulators (RRs)	*rr01*	1	1	1	1	1	1	1	1	1	1	1	1	1	1	1	1	1	1	1	1	1	1	1	1	1
	*rr02*	1	1	1	1	1	1	1	1	1	1	1	1	1	1	1	1	1	1	1	1	1	1	1	1	1
	*rr03*	1	1	1	1	1	1	1	1	1	1	1	1	1	1	1	1	1	1	1	1	1	1	1	1	1
	*rr04*	1	1	1	1	1	1	1	1	1	1	1	1	1	1	1	1	1	1	***1***	1	1	1	1	1	1
	*rr05*	1	1	1	1	1	1	1	1	1	1	1	1	1	1	1	1	1	1	1	1	1	1	1	1	1
	*rr06*	1	1	1	1	1	1	1	1	1	1	1	1	1	1	1	1	1	1	1	1	1	1	1	1	1
	*rr07*	1	1	1	1	1	1	1	1	1	1	1	1	1	1	1	0	1	1	1	1	1	1	1	1	1
	*rr08*	1	1	1	1	1	1	1	1	1	1	1	1	1	1	1	1	1	1	1	1	1	1	1	1	1
	*rr09*	1	1	1	1	1	1	1	1	1	1	1	1	1	1	1	1	1	1	1	1	1	1	1	1	1
	*rr10*	1	1	1	1	1	1	1	1	1	1	1	1	1	1	1	1	1	1	1	1	1	1	1	1	1
	*rr11*	1	1	1	1	1	1	1	1	1	1	1	1	1	1	1	1	1	1	1	1	1	1	1	1	1
	*rr12*	1	1	1	1	1	1	1	1	1	1	1	1	1	1	1	1	1	1	1	1	0	1	1	1	1
	*rr13*	1	1	1	1	1	1	1	1	1	1	1	1	1	1	1	1	1	1	1	1	1	1	1	1	1
	*rr14*	1	1	1	1	1	1	1	1	1	1	1	1	1	1	1	1	1	1	1	1	1	**2**	1	1	1

The table shows, the absence (0) or presence (1 or 2) of each gene considered in the 25 strains selected for this study. The number (1 or 2) indicates the amount of copies per gene in each genome. *rr14* is the only gene with more than one copy per genome (**2**), which is actually fused with its neighbor gene (*gnd*) upstream. In bold and italics, three genes are observed (*hk01*, *hk12* and *rr04*) in two different strains which might have some alteration (insertion or deletion) in their primary DNA and protein Sequence. The two-component system NisK-NisR of the pneumococcus is rare, of the 25 strains analyzed in this study only in the strain 70,585 was found

The estimation of the variability for each individual virulence factor and pneumococcal regulator (at the DNA and protein level) allowed the construction of a partial variome for the analysed 25 pneumococcal strains. Briefly, the variome takes into consideration the estimation of (1) the presence, absence or the number of copies of genes in the different strains, (2) the number of total synonymous and nonsynonymous mutations, and (3) the number of allelic and protein variants explaining the variability for each factor. The results summarized in Tables 5 and 6, contain the data for the genes and proteins associated to virulence and host-pathogen interaction, and also the data for the stand-alone and TCS regulators. Specifically there are some identified factors with the best distribution and highest evolutionary conservation, These were (1) the *ply* gene encoding the sole pneumococcal cytolysin and cytotoxin pneumolysin [46], (2) the *enolase*, which encodes the enzyme enolase (2-phosphoglycerate dehydratase) and has an essential function in the metabolism [47], but also interacts specifically with plasmin(ogen) and is therefore involved in fibrinolytic processes, adherence and virulence, and (3) the *pcsB* (Usp45) gene, which encodes for a 45-kDa secreted and immunogenic protein that is involved in cell division and stress response [48]. As for the mutations, these three proteins presented a minor number of changes, in comparison with others proteins that were also analyzed. The variome of the TCS (Table 6) allowed to conclude that the most conserved genes from the evolutionary point of view, are the genes *hk05* and *rr05 of ciaR/H (tcs05)*. The TCS CiaRH is involved in the resistance to cefotaxime, regulation of genetic competence and increase in pathogenicity in the respiratory tract in murine models [7, 49, 50]. Meanwhile, *hk02* and *rr02 (WalR/K, MicA/B or VicR/K),* have been associated with resistance to erythromycin and are essential for the bacterial growth. Nevertheless, the latter was proven to be due to its regulon (*pcsB*), and was no longer essential upon ectopic expression of PcsB [7, 48]. Pneumococcal TCS08 is involved in the genetic regulation of pilus-1 [41]. The mutation analysis showed that the response regulators exhibited a lower rate of variations in comparison to the histidine kinases, being the response regulators *rr05*, *rr02*, *rr06*, and *rr08* the most conserved. All the results obtained in this study support the global idea of a new generation of protein-based and serotype-independent vaccines for *Streptococcus pneumoniae*. The basis is the high degree of distribution and conservation of the virulence proteins in combination with the importance of their functions and immunogenic capacities. This probably makes them ideal pharmacological targets to treat the pneumococcus and its diseases. This might be an alternative to the immunization with the conjugated serotypes, or represent a strategy to combine immunogenic and highly conserved proteins with capsular polysaccharides to generate a serotype-independent immune response.

**Table 5:** Analysis of the Variome of the virulence factor genes of *S. pneumoniae*

Virulence Factor Genes		Length		Mutations			Variants		Analyzed Sequences
Name	Locus in TIGR4 / R6	Gene (bp)	Protein (a.a.)	Overall	Synonimous	Non-Synonimous	Allelles	Protein	
*lytA*	*sp_1937 / spr1754*	957	318	208	154	54	25	20	42
*gnd*	*sp_0375 / spr0335*	1425	474	82	75	7	19	11	26
*strH*	*sp_0057 / spr0057*	3939	1312	87	32	55	21	21	25
*lytB*	*sp_0965 / spr0867*	1977	658	132	84	48	22	20	25
*endoD*	*sp_0498 / spr0040*	4980	1659	139	70	69	20	20	25
*nanA*	*sp_1693 / spr1536*	3108	1035	460	282	178	20	19	25
*bgaA*	*sp_0648 / spr0565*	6702	2233	415	247	168	19	18	25
*spGH101*	*sp_0368 / spr0328*	5304	1767	348	238	110	18	18	25
*glnQ*	*sp_0609 / spr0534*	765	254	40	22	18	17	17	25
*phtE*	*sp_1004 / spr0908*	3120	1039	56	28	28	20	16	25
*pbp1B*	*sp_2099 / spr1909*	2466	821	81	16	65	17	16	25
*pce*	*sp_0930 / spr0831*	1884	627	148	80	68	16	16	25
***pavA***	*sp_0966 / spr0868*	1656	551	40	18	22	17	**15**	**25**
***cbpD***	*sp_2201 / spr2006*	1347	448	49	28	21	18	**14**	**25**
***piuA***	*sp_1872 / spr1687*	966	321	22	7	15	14	**12**	**25**
***htrA***	*sp_2239 / spr2045*	1182	393	12	8	4	14	**10**	**25**
***pclA***	*sp_1546 / spr1402*	630	209	19	9	10	13	**10**	**25**
***mgrA***	*sp_1800 / spr1622*	1482	493	21	15	6	13	**9**	**25**
***ppiA***	*sp_0771 / spr0679*	804	267	16	8	8	12	**9**	**25**
***stkP***	*sp_1732 / spr1577*	1980	659	52	46	6	18	**7**	**25**
***gap***	*sp_2012 / spr1825*	1008	335	14	12	2	13	**7**	**25**
***psaA***	*sp_1650 / spr1494*	930	309	53	41	12	13	**6**	**25**
***psaR***	*sp_1638 / spr1480*	651	216	10	5	5	9	**6**	**25**
***piaA***	*sp_1032 / spr0934*	1026	341	9	2	7	7	**6**	**25**
***usp45***	*sp_2216 / spr2021*	1179	392	9	6	3	8	**4**	**25**
***eno***	*sp_1128 / spr1036*	1305	434	17	16	1	12	**2**	**25**
*spuA*	*sp_0268 / spr0247*	3843	1280	121	77	44	18	18	24
*prtA*	*sp_0641 / spr0561*	6423	2140	436	272	164	18	16	24
*nanB*	*sp_1687 / spr1531*	2094	697	45	20	25	18	16	24
*pfbA*	*sp_1833 / spr1652*	2127	708	209	80	129	15	15	24
*pppA*	*sp_1572 / spr1430*	537	178	69	43	23	16	12	24
***ply***	*sp_1923 / spr1739*	1416	471	20	19	1	14	**2**	**24**
*pavB*	*sp_0082 / spr0075*	2574	857	(−)	(−)	(−)	20	17	23
*phtD*	*sp_1003 / spr0907*	2520	839	604	331	273	17	17	23
*zmpB*	*sp_0664 / spr0581*	5646	1881	(−)	(−)	(−)	15	15	23
*pspA*	*sp_0117 / spr0121*	2235	744	(−)	(−)	(−)	18	17	22
*cbpC*	*sp_0377 / spr0337*	1023	340	176	87	89	15	14	22
*ksgA*	*sp_1992 / spr1806*	666	221	36	8	28	14	14	22
*ppmA*	*sp_0981 / spr0884*	942	313	9	6	3	9	6	22
*hysA*	*sp_0314 / spr0286*	3201	1066	91	41	50	18	17	20
*lytC*	*sp_1573 / spr1431*	1473	490	80	20	60	17	16	20
*pspC*	*sp_2190 / spr1995*	2082	693	(−)	(−)	(−)	19	19	19
*aliA*	*sp_0366 / spr0327*	1986	661	67	50	17	17	17	19
*SP_0667*	*sp_0667 / spr0583*	999	332	87	49	38	13	13	19
*merR*	*sp_0739 / spr0649*	741	246	17	9	8	12	9	19
*pcpA*	*sp_2136 / spr1945*	1866	621	43	25	18	17	13	18
*SP_1492*	*sp_1492 / spr1345*	609	202	18	6	12	11	11	18
*cbpG*	*sp_0390 / spr0349*	858	285	106	47	59	15	15	17
*zmpA*	*sp_1154 / spr1042*	6015	2004	(−)	(−)	(−)	10	10	17
*cbpJ*	*sp_0378 / Absent*	999	332	87	52	35	10	9	15
*nanC*	*sp_1326 / Absent*	2223	740	64	39	25	7	7	10
*phtA*	*sp_1175 / spr1061*	2409	802	48	34	14	8	7	9
*phtB*	*sp_1174 / Absent*	2460	819	331	202	129	7	7	8
*cbpF*	*sp_0391 / spr0351*	1023	340	57	29	28	7	7	8
*zmpD*	*Absent / Absent*	5238	1745	(−)	(−)	(−)	6	5	7
*rrgA*	*sp_0462 / Absent*	2682	893	420	225	195	5	5	7
*rrgC*	*sp_0464 / Absent*	1182	393	19	8	11	5	5	7
*rrgB*	*sp_0463 / Absent*	1998	665	738	290	448	4	4	7
*rlrA*	*sp_0461 / Absent*	1530	509	0	0	0	2	2	7
*pitA*	*Absent / Absent*	1770	589	1	0	1	5	5	6
*pitB*	*Absent / Absent*	1233	410	0	0	0	3	3	6
*psrP*	*sp_1772 / Absent*	14,331	4776	(−)	(−)	(−)	5	5	5
*zmpC*	*sp_0071 / Absent*	5571	1856	2	1	1	2	2	3
*cbpI*	*sp_0069 / Absent*	636	211	0	0	0	1	1	3

Data of the punctual genetic variability (total mutations, synonymous and nonsynonymous + allelic and protein variants) estimated for each one of the virulence factors and simple regulators genes. The analyzed sequences depend on the presence, absence or number of copies of the genes in the different strains. The size of the sequences and loci are also shown in TIGR4 and R6, pneumococcal representative strains. Factors in bold were identified as the most conserved. (−) = mutations could not be estimated for different reasons, like repetitive sequences

**Table 6:** Analysis of the genetic variation (Variome) of the genes that conform the two-component systems in *S. pneumoniae*

Virulence Factor Genes		Length		Mutations			Variants		Analyzed Sequences
Name	Locus in TIGR4 / R6	Gene (bp)	Protein (a.a.)	Overall	Synonimous	Non-Synonimous	Allelles	Protein	
*rr14*	*sp_0376 / spr0336*	690	229	17	15	2	14	5	26
*hk11*	*sp_2001 / spr1815*	1098	365	247	142	105	18	18	25
*hk10*	*sp_0604 / spr0529*	1329	442	33	16	17	17	16	25
*hk06*	*sp_2192 / spr1997*	1332	443	33	23	10	17	12	25
*hk13*	*sp_0527 / spr0464*	1341	446	272	153	119	12	11	25
*rr13*	*sp_0526 / spr0463*	738	245	78	65	13	11	11	25
*rr11*	*sp_2000 / spr1814*	600	199	77	56	21	15	10	25
*hk03*	*sp_0386 / spr0343*	996	331	36	26	10	13	10	25
*hk01*	*sp_1632 / spr1473*	975	324	20	11	9	13	10	25
*hk09*	*sp_0662 / spr0579*	1692	563	23	17	6	15	8	25
*rr01*	*sp_1633 / spr1474*	678	225	13	10	3	13	8	25
*rr09*	*sp_0661 / spr0578*	738	245	14	10	4	12	7	25
*rr04*	*sp_2082 / spr1893*	708	235	49	17	32	9	6	25
*rr03*	*sp_0387 / spr0344*	633	210	14	10	4	12	5	25
*rr10*	*sp_0603 / spr0528*	657	218	15	10	5	11	5	25
*rr06*	*sp_2193 / spr1998*	654	217	9	5	4	7	5	25
***rr08***	*sp_0083 / spr0076*	699	232	10	6	4	10	**4**	**25**
***hk04***	*sp_2083 / spr1894*	1332	443	12	9	3	10	**4**	**25**
***hk08***	*sp_0084 / spr0077*	1053	350	14	12	2	11	**3**	**25**
***rr05***	*sp_0798 / spr0707*	675	224	6	5	1	11	**3**	**25**
***hk02***	*sp_1226 / spr1106*	1350	449	12	10	2	10	**3**	**25**
***rr02***	*sp_1227 / spr1107*	705	234	7	7	0	11	**2**	**25**
***hk05***	*sp_0799 / spr0708*	1335	444	11	10	1	9	**2**	**25**
*hk07*	*sp_0155 / spr0153*	1647	548	68	46	22	19	17	24
*rr07*	*sp_0156 / spr0154*	1287	428	50	33	17	17	14	24
*rr12*	*sp_2235 / spr 2041*	753	250	11	8	3	10	3	24
*hk12*	*sp_2236 / spr2042*	1326	441	42	16	26	11	9	23

Data of the punctual genetic variability (total mutations, synonymous and non-synonymous + allelic and protein variants) estimated for each one of the two-component system genes. The analyzed sequences depend on the presence, absence or number of copies of the genes in the different strains. The size of the sequences and loci are also shown in TIGR4 and R6, pneumococcal representative strains. Factors in bold are the most conserved

## Conclusions

The construction of this “low-scale” Variome model for the virulence factors and regulators of *Streptococcus pneumoniae* was achieved from 25 pneumococcal strains with fully sequenced and annotated genomes. According to the Molecular Phylogenetic Analysis performed on the NCBI website, this selected set of pneumococcal genomes ensured an optimal representation of the pneumococcal population (8290 strains) reported in the NCBI database up to date. Similarly, this study population set also represented an important group of highgly pathogenic pneumococcal serotypes (1, 2, 3, 4, 5, 6B, 11A, 14, 19A, 19F and 23F), which have been also included in the current pneumococcal conjugate vaccine formulations (except serotypes 2 and 11A), used to prevent penumococal infections. A total of 92 different genes and proteins were identified, classified, and studied for the construction of the variome. The genes of the pneumococcal virulence factors and TCS, are distributed along the genome, and are located in such a manner that transcription is co-oriented with replication. The analysis of the gene distribution in this study population set showed that 26 of them were found in the 100% of the 25 pneumococcal genomes/strains (core genome), while 39 are part of the flexible genome. The estimation of the variability for each individual virulence factors, stand-alone regulator or TCS, indicated that the virulence factors with the lowest variability in the pneumococcus are pneumolysin, enolase and PcsB, while the regulators with the highest conservation are TCS05 (CiaR/H), TCS02 (VicR/K) and TCS08. Finally, all the results obtained here with the bioinformatic analysis performed, constitute the first model to compare, visualize and understand the future flood of new genomic data about the genetic variation (in terms of gene presence/absence or mutation) of pneumococcal virulence factors and regulators [51–53]. The applicability offered by this variome model, together with further population genomic analysis of pneumococci, will provide relevant information on potential targets for vaccines, supporting the idea of a new generation of protein-based formulations to combat *Streptococcus pneumoniae* and its disease burden.

## Abbreviations

BLAST: Basic local alignment search tool
BLOSUM: Blocks substitution matrix
CBPs: Choline binding proteins
CSF: Cerebrospinal fluid
DnaSP: DNA sequence polymorphism
HK: Histidine kinase
IPDs: Invasive pneumococcal diseases
KEGG: Kyoto encyclopedia of genes and genomes
MultAlin: Multiple sequence alignment
NCBI: National center for biotechnology information
NCSP: Non-classical surface proteins
P2CS: Prokaryotic two-component systems
PAI: Pathogenicity Islands
RR: Response regulator
*S. p.*: *Streptococcus pneumoniae*
TCS: Two-Component regulatory Systems
UNIPROT: The Universal Protein Resource
Variome: Pan-genomic variability map
VFDB: Virulence factors data base
